# Central neuroendocrine dysregulation in ischaemic stroke sequelae: pathophysiological mechanisms—a narrative review

**DOI:** 10.3389/fnhum.2026.1836343

**Published:** 2026-07-23

**Authors:** Han Gong, Dan Liu, Xiang-Zheng Wang, Jia-Sheng Rao, Xiao-Xia Du

**Affiliations:** 1 Beijing Key Laboratory for Biomaterials and Neural Regeneration, National Medical Innovation Platform for Industry-Education Integration in Advanced Medical Devices (Interdiscipline of Medicine and Engineering), School of Biological Science and Medical Engineering, Beihang University, Beijing, China; 2 China Rehabilitation Research Center, Beijing, China; 3 China Rehabilitation Science Institute, Beijing, China; 4 School of Rehabilitation Sciences and Engineering, University of Health and Rehabilitation Sciences, Qingdao, China; 5 Beijing International Cooperation Bases for Science and Technology on Biomaterials and Neural Regeneration, Beijing Advanced Innovation Center for Biomedical Engineering, Beihang University, Beijing, China; 6 School of Rehabilitation, Capital Medical University, Beijing, China; 7 School of Rehabilitation Medicine, Wenzhou Medical University, Wenzhou, China

**Keywords:** glucocorticoids, hypothalamic–pituitary–adrenal axis, ischemic stroke, neuroendocrine-immune interactions, neurological dysfunction

## Abstract

Ischemic stroke (IS) prognosis is frequently compromised by secondary systemic and neuropsychiatric complications extending beyond the initial brain injury. The hypothalamic–pituitary–adrenal (HPA) axis, the primary neuroendocrine regulator, plays a pivotal yet underappreciated role in the pathogenesis of these sequelae. This review analyzes the adverse impact of HPA axis dysregulation on post-stroke outcomes across disease phases. In the acute phase, maladaptive glucocorticoid surges and circadian disruption drive metabolic disturbances, blood–brain barrier (BBB) breakdown leading to hemorrhagic transformation, and stroke-induced immunodepression that predisposes patients to pneumonia. In the chronic phase, persistent HPA hyperactivity, driven by impaired negative feedback and glucocorticoid receptors (GRs) resistance, induces structural remodeling in emotional and cognitive circuits. This serves as a shared pathophysiological mechanism underpinning post-stroke depression, anxiety, and cognitive impairment. Consequently, elucidating these neuroendocrine-immune interactions provides a novel pathophysiological framework for understanding post-stroke multidimensional complications.

## 1 Introduction

Ischemic stroke (IS) ranks as the second leading cause of death and a primary cause of long-term disability worldwide, characterized by its high incidence, recurrence, and morbidity rates (Lian et al., 2025). IS is precipitated by the stenosis or occlusion of cerebral arteries, which results in a critical reduction of cerebral blood flow. This hemodynamic failure induces focal ischemia and hypoxia, ultimately culminating in neuronal necrosis and the rapid loss of neurological functions governed by the affected regions. While modern medical management has successfully lowered mortality rates, many survivors are left with varying degrees of neurological deficits. These impairments often manifest as motor, sensory, cognitive, or linguistic dysfunction. Collectively, they represent a major challenge and impose a profound burden on global public health (Han et al., 2025).

While reperfusion therapies are widely applied, the risk of recurrence remains high, and survivors frequently endure persistent functional sequelae. Beyond the local metabolic imbalance caused by the acute interruption of cerebral blood supply, ischemia triggers complex pathological cascades involving excitotoxicity, neuroinflammation, oxidative stress, and blood–brain barrier (BBB) disruption, which collectively drive secondary brain injury (Candelario-Jalil et al., 2022). Notably, growing evidence suggests that these pathological processes are not merely isolated events confined to the brain, but act as systemic triggers interacting with neuroendocrine stress responses (Balch et al., 2020). Specifically, the hypothalamic–pituitary–adrenal (HPA) axis, acting as a bridge between the central nervous system (CNS) and peripheral physiology, plays a central role in regulating the adaptive response to ischemic injury.

Upon the onset of an IS event, the HPA axis is rapidly activated as an integral component of the systemic stress response, precipitating a cascading release of corticotropin-releasing hormone (CRH), adrenocorticotropic hormone (ACTH), and glucocorticoids (GCs)—specifically cortisol in humans and corticosterone in rodents. The resulting sustained release of GCs facilitates their binding to GRs located on vascular endothelial cells, glial cells, and neurons. This interaction exerts a dual regulatory effect dictated by strict dose and temporal boundaries. During the hyperacute window, transient physiological GC elevations confer neuroprotection by maintaining cerebral hemodynamics and restraining early excessive inflammation. Conversely, prolonged exposure to supraphysiological GC levels during the subacute and chronic phases transitions into a neurotoxic driver of post-ischemic injury (Zucchi et al., 2010; Williams and Ghosh, 2020).

During the acute phase of ischemic stroke (AIS), sustained HPA axis hyperactivation is implicated in systemic metabolic dysregulation and immune dysfunction (Shimba et al., 2018). Furthermore, it exacerbates post-stroke neuroinflammation and modulates post-injury neuroplasticity (Hapgood et al., 2016). Mechanistically, GC-induced hepatic gluconeogenesis and peripheral insulin resistance drive stress hyperglycemia (Cho and Suh, 2024; Zhou et al., 2024). Observed in up to 60% of AIS patients, this phenomenon aggravates infarct expansion and is predictive of poor functional recovery (Li and Cummins, 2022).

In the chronic phase of IS, sustained hyperactivity of the HPA axis has been identified as a robust predictor of post-stroke depression (PSD) and vascular cognitive impairment (VCI). Specifically, elevated nocturnal cortisol levels are independently associated with hippocampal atrophy and executive dysfunction (Juruena et al., 2004; Shimba and Ikuta, 2020). Immunologically, chronic exposure to excessive GCs paradoxically polarizes microglia toward a pro-inflammatory sensitized state. This sensitization renders brain tissue susceptible to secondary inflammatory insults, perpetuating a self-reinforcing cytokine storm that exacerbates BBB disruption and neuronal injury (Kim et al., 2022).

These HPA-mediated pathways converge to form a maladaptive positive feedback loop: PSD and cognitive decline reduce treatment adherence, stress hyperglycemia impairs neuroplasticity, and uncontrolled inflammation accelerates atherosclerosis progression—collectively amplifying the risk of recurrence and chronic disability (Kim et al., 2022). These alterations in neuroendocrine regulation are consistently linked to deficits in memory, executive function, and emotional regulation, suggesting that stress response dysregulation is not merely an epiphenomenon of stroke but likely a direct participant in disease progression by compromising neural circuit integrity in critical regions such as the hippocampus and prefrontal cortex (PFC).

As the central regulatory system of the physiological stress response, the functional status of the HPA axis is closely correlated with the disease progression and prognosis of IS. Existing literature exploring the role of the HPA axis in stroke pathophysiology has expanded our understanding of this field to varying degrees. Specifically, prior investigations have quantified the correlation between acute-phase cortisol levels and early clinical mortality (Barugh et al., 2014), delineated the link between localized neuroanatomical damage and aberrant HPA axis activation (Gulyaeva et al., 2021), and systematically reviewed the profound impacts of hypercortisolemia on post-stroke cognitive and emotional disorders (Wang et al., 2024). Additionally, insights from hyperacute interventions suggest that the corticotropin-releasing factor (CRF) peptide family modulates the stress response to confer early neuroprotection (Lichlyter et al., 2023).

Although accumulating evidence links sustained HPA axis activation to adverse neurological outcomes, the global role of HPA axis dysregulation within the post-stroke disease trajectory has yet to be systematically integrated. Current investigations remain predominantly restricted to isolated pathological pathways or single clinical outcomes. The HPA axis functions as a central regulatory network to orchestrate multi-system complications. Elucidating this role remains an unfinished task, as the underlying mechanistic crosstalk bridging post-stroke neuropsychiatric disorders, metabolic disturbances, and immune-inflammatory imbalances has yet to be fully clarified. Meanwhile, therapeutic strategies targeting HPA axis dysfunction remain largely overlooked within existing clinical management paradigms.

**Figure 1:**
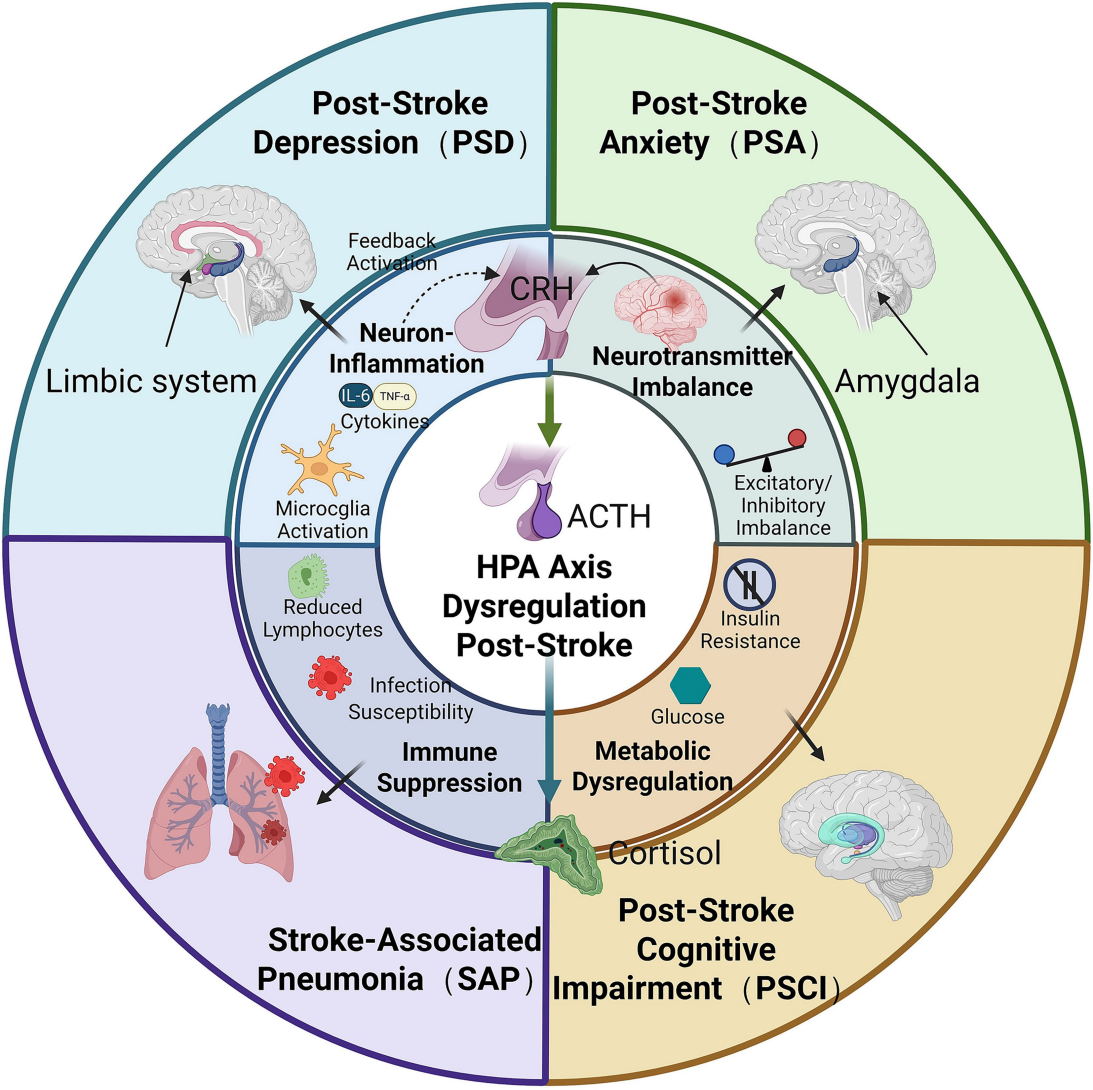
Multidimensional complications driven by post-stroke HPA axis dysregulation. Following ischemic stroke, the HPA axis is overactivated, characterized by the central cascade of CRH, ACTH, and cortisol, which subsequently initiates a spectrum of central and peripheral complications. Top left: Neuroinflammation in the limbic system is exacerbated, marked by microglia activation and the release of cytokines, leading to PSD and further modulated by feedback mechanisms. Top right: Neurotransmitter homeostasis in the amygdala is disrupted, creating an E/I imbalance that drives PSA. Bottom right: Metabolic dysregulation is induced by systemic cortisol elevation, primarily manifesting as insulin resistance and abnormal glucose metabolism, contributing to PSCI. Bottom left: Profound immune suppression is caused by peripheral hypercortisolism, evidenced by reduced lymphocyte counts and heightened infection susceptibility, culminating in SAP. Created in https://BioRender.com.

To address this gap, the present review comprehensively outlines the pathophysiological evolution of the HPA axis following IS. On this basis, we focus on dissecting the mechanistic interactions among HPA axis dysfunction, neurological deficits, and multi-system complications. By cross-examining clinical and preclinical evidence, we re-evaluate the post-stroke disease trajectory from a neuroendocrine perspective. This approach characterizes HPA axis dysfunction as a critical hub linking neuropsychiatric, metabolic, and immune-inflammatory sequelae. In this review, the pathological alterations of the post-stroke HPA axis are correlated with stroke-induced complications to analyze how neuroendocrine dysregulation mediates these secondary outcomes (see Figure 1).

## 2 Ischemic cascade triggering HPA axis activation

The pathophysiology of IS is a complex and dynamic cascade involving multiple interrelated pathological processes, including (1) disrupted energy metabolism and ionic homeostasis, (2) excitotoxicity and calcium overload, (3) blood–brain barrier disruption and neuroinflammation, and (4) ischemia–reperfusion injury. These pathological changes are highly interconnected rather than occurring independently, collectively generating profound neurochemical and metabolic stress signals that stimulate the paraventricular nucleus (PVN) of the hypothalamus and subsequently drive the hyperacute activation of the HPA axis.

After IS, blood flow in the ischemic core region is interrupted, leading to cell death supplied by this blood supply and resulting in irreversible necrosis of brain tissue. Simultaneously, a penumbra surrounds this necrotic core; this region represents hypoperfused tissue where cellular activity is suppressed due to bioenergetic compromise, yet structural integrity is transiently preserved (Astrup et al., 1981; Dirnagl et al., 1999). However, in the absence of reperfusion, neuronal necrosis progresses rapidly.

As ATP stores are depleted, the functional integrity and expression of ATP-dependent ion pumps, notably the Na+/K+ -ATPase, are severely impaired (Simard et al., 2007). This bioenergetic failure disrupts the transmembrane concentration gradients of Na+/K+ -ATPase, and H+. Consequently, the resulting osmotic imbalance drives a massive influx of interstitial fluid into the intracellular space, culminating in cytotoxic edema (Stokum et al., 2016; O’Donnell, 2014; Kahle et al., 2009). The disruption of transmembrane Na+ and K+ gradients induces sustained membrane depolarization, which triggers the opening of voltage-gated Ca2+ channels (VGCCs) and consequently precipitates a massive exocytotic release of glutamate from presynaptic terminals, resulting in aberrant neuronal hyperexcitability (Anwar et al., 2025). This receptor activation facilitates a massive Ca2+ influx that overwhelms intracellular buffering capacity, leading to excitotoxicity and cytosolic calcium overload (Khan et al., 2025). The resulting intracellular Ca2+ surge activates protein kinases such as CaMKII and PKC, as well as proteases, thereby exacerbating neuronal hyperexcitability and ultimately triggering necrotic and apoptotic cell death pathways.

The BBB is pivotal for maintaining CNS homeostasis and protecting neural tissue (Castillo-González and González-Rey, 2025). Following cerebral hypoxia, cerebrovascular endothelial dysfunction increases inflammatory mediator release and induces vasogenic edema, resulting in altered BBB permeability, which has been identified as an independent predictor of poor motor and cognitive outcomes (Lacoste et al., 2025; Zi and Shuai, 2013). Stroke triggers the release of large amounts of pro-inflammatory mediators, while persistent immune responses are observed during both the acute and chronic stages (Shen et al., 2019; Maziz et al., 2025). Cerebral ischemia and hypoxia further induce excessive ROS production, initiating inflammatory cascades that promote mitochondrial apoptosis, disrupt cellular energy metabolism, and ultimately lead to cell death (Meng et al., 2025; Cheng et al., 2022). In addition, interactions between central and peripheral immune responses aggravate BBB disruption after ischemic stroke, forming a positive feedback inflammatory loop that exacerbates secondary brain injury (Chen et al., 2019; Cho et al., 2022). This pathological cascade, termed cerebral ischemia–reperfusion injury (CIRI), causes secondary damage to the brain, thereby exacerbating the aforementioned pathophysiological processes and worsening post-stroke functional outcomes (Zhang et al., 2022).

In summary, following ischemic stroke onset, the affected brain tissue undergoes multiple interacting pathophysiological processes, including energy metabolic failure, excitotoxicity, oxidative stress, and neuroinflammation, which collectively contribute to BBB disruption and cerebral edema. This localized microenvironmental derangement and the accumulated inflammatory mediators serve as potent neural and humoral stress signals that breach or bypass compromised central boundaries, thereby stimulating the PVN of the hypothalamus and initiating the hyperacute activation of the HPA axis.

## 3 Physiological role of the HPA axis

Internal homeostasis is maintained by the dynamic equilibrium of hormonal levels, with the regulation of the HPA axis serving as a critical mechanism in this process. As a fundamental component of the neuroendocrine system, the HPA axis orchestrates the physiological stress response and operates under precise negative feedback control, thereby playing a pivotal role in preserving endocrine stability (Herman et al., 1996).

### 3.1 Initiation and neurotransmitter regulation of the HPA axis

The HPA axis cascade is initiated within the PVN of the hypothalamus. Parvocellular neurons in the PVN synthesize and secrete arginine vasopressin (AVP) and CRH (Herman et al., 2020). CRH stimulates the anterior pituitary to secrete ACTH and, as the principal hypothalamic regulator, exerts context-dependent effects on neurovascular function following ischemic stroke (Deussing and Chen, 2018; Lichlyter et al., 2023). The integration of these signals within the PVN is tightly regulated by central neurotransmitters.

*γ*-Aminobutyric acid (GABA) is the principal inhibitory neurotransmitter in the central nervous system. GABAergic neurons play a critical role in modulating HPA axis activity by directly inhibiting PVN efferent impulses and attenuating ACTH secretion. Conversely, glutamate exerts an activating effect on the HPA axis. These inhibitory and excitatory projections to the PVN are governed by descending inputs from limbic forebrain structures. The balance between GABAergic and glutamatergic signaling plays a pivotal role in the central integration of the HPA stress response (Herman et al., 2004). Collectively, the interaction between these neurotransmitter circuits and PVN neurons determines the activation threshold of the HPA axis.

### 3.2 Effector hormones and physiological functions

ACTH induces adrenocortical synthesis and secretion of GCs. GCs exert their physiological effects by binding to intracellular GRs (Vandevyver et al., 2014). Key metabolic outcomes include promoting hepatic glycogenolysis and gluconeogenesis, which results in elevated blood glucose levels. GCs exhibit a critical dose-dependent effect on neurological health. Under physiological conditions, GCs are indispensable for maintaining cognitive function. Conversely, chronic exposure to supraphysiological cortisol levels—as reflected in the brain and cerebrospinal fluid (CSF)—is associated with numerous adverse neurological conditions, specifically declarative memory impairment and cognitive decline (Ouanes et al., 2017).

Furthermore, it is noteworthy that cortisol synthesis and secretion exhibit a robust circadian rhythm, typically characterized by a morning acrophase and a nocturnal nadir. Controlled by the hypothalamic suprachiasmatic nucleus (SCN), this rhythm ensures that GC levels peak during the active phase and decline during the resting phase. GCs are steroid hormones produced by the adrenal cortex under the dual regulation of circadian rhythms and stress. These circadian factors collectively orchestrate the diurnal oscillations of innate and adaptive immunity. GCs drive these oscillations by regulating T-cell distribution via the induction of IL-7 and CXCR4 signaling (Shimba et al., 2018). Consequently, GCs modulate the circadian rhythms of innate and adaptive immunity through bidirectional regulatory effects (Shimba and Ikuta, 2020). Reciprocally, immune cells exert feedback regulation on the HPA axis; pro-inflammatory cytokines such as TNF-*α*, IL-1, and IL-6 can activate CRH signaling at the level of the PVN, thereby stimulating downstream ACTH and GC release. In turn, GCs exert potent anti-inflammatory effects. Therefore, sustained dysregulation of GC release disrupts immune rhythmicity, leading to maladaptive inflammation (Coutinho and Chapman, 2011; Cain and Cidlowski, 2017). Additionally, the circadian cycling of GCs contributes to metabolic regulation, participating in hepatic glycogenesis and adipose tissue lipolysis (Kuo et al., 2015).

### 3.3 Negative feedback mechanisms of the HPA axis

Under physiological conditions, elevated GC levels exert a negative feedback effect on the hypothalamus and pituitary to inhibit their activity. This mechanism constitutes the cornerstone of maintaining neuroendocrine homeostasis. GC-mediated negative feedback occurs not only at the level of the hypothalamic PVN and anterior pituitary (short-loop feedback) but relies heavily on long-loop feedback mediated by the limbic system. The hippocampus and medial PFC (mPFC) are key brain regions enriched with corticosteroid receptors that exert tonic inhibition on the PVN via polysynaptic pathways (Herman et al., 2016; Herman et al., 1996; Ulrich-Lai and Herman, 2009). This inhibitory control is essential for preventing aberrant HPA axis activation under non-stress conditions.

The efficiency of this feedback is coordinately determined by mineralocorticoid receptors (MRs) and GRs. Interestingly, this regulation exhibits circadian dependence. Specifically, MRs possess high affinity for cortisol and are predominantly occupied at nadir levels. In contrast, GRs have low affinity and are activated only during stress-induced hypercortisolemia or circadian peaks (de Kloet et al., 2005). Therefore, the post-stroke surge in GCs primarily initiates the termination of the stress response by activating GR signaling pathways. The hippocampus and PFC exhibit the highest expression densities of both corticosteroid receptor types, rendering them highly susceptible to this MR/GR stoichiometric imbalance (Popoli et al., 2011). Within these specific neural microenvironments, sustained GR overactivation suppresses long-term potentiation (LTP) and drives dendritic spine retraction. This receptor mismatch alters the structural integrity of local synaptic circuits under conditions of prolonged glucocorticoid elevation. Chronic neuroinflammation and prolonged glucocorticoid exposure may progressively induce GR resistance within the hippocampus and PFC, thereby weakening negative feedback control of the HPA axis. This impairment contributes to persistent hypercortisolemia and establishes a self-perpetuating cycle linking neuroendocrine dysfunction with cognitive and emotional sequelae (Juruena, 2026).

In summary, HPA axis activation elicits a broad spectrum of effects across the cardiovascular, metabolic, and immune systems (Herman and Cullinan, 1997).

## 4 Pathophysiological alterations of the HPA axis post-stroke

Acute ischemic stroke constitutes a profound physiological stressor, leading to activation of the HPA axis, in part through sympathetic nervous system (SNS) engagement (Fassbender et al., 1994). Studies utilizing the middle cerebral artery occlusion (MCAO) model have identified excessive glutamate release as an early driving factor in stress axis activation. Glutamate is considered a key mediator of HPA axis hyperactivation under ischemic conditions (You et al., 2018). Specifically, He et al. Investigated the temporal dynamics of glutamate release and HPA axis activity following MCAO in rats (He et al., 2003). Their findings demonstrated that glutamate levels in the hippocampus and hypothalamus increased rapidly, reaching peak concentrations within 15 min after ischemia onset. Concurrently, plasma ACTH levels were significantly elevated during the same time window. During the peak phase of reperfusion injury, hypothalamic glutamate levels were positively correlated with corticotropin-releasing CRH mRNA expression.

Concurrently, the post-stroke environment is characterized by a significant upregulation of pro-inflammatory cytokines, including IL-1β, TNF-*α*, and IL-6. These mediators synergistically stimulate CRH and ACTH secretion. Furthermore, this inflammatory milieu compromises GC negative feedback loops through aberrant GR phosphorylation and the induction of transcriptionally inactive GR isoforms (Barthels and Das, 2020). Supporting this interaction, Onufriev et al. demonstrated a concurrent accumulation of corticosterone and IL-1*β* in both the ipsilateral and contralateral hippocampus and frontal cortex (FC) in MCAO model rats (Onufriev et al., 2022). Furthermore, AVP, synthesized in the hypothalamus, acts synergistically with CRH. This synergy regulates corticosteroid secretion within the central nervous system and periphery under post-stroke stress conditions. By facilitating exaggerated ACTH release, AVP drives the sustained adrenocortical secretion of GCs, thereby potentiating the endocrine stress response (Fassbender et al., 1994).

**Figure 2:**
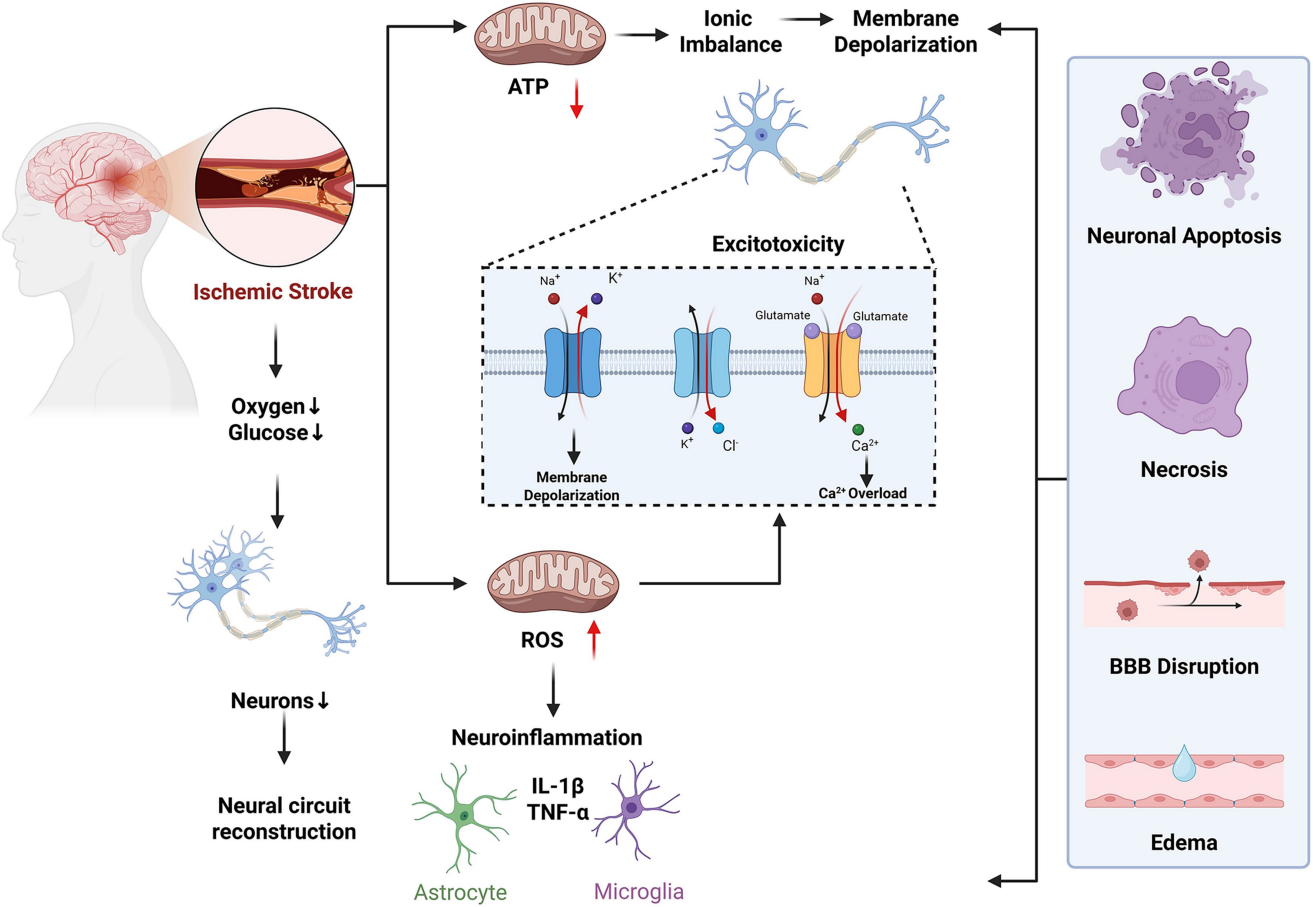
Schematic diagram of the pathophysiological cascade following ischemic stroke. Ischemia-induced oxygen and glucose deprivation causes mitochondrial dysfunction, resulting in ATP depletion and excessive ROS generation. Energy failure subsequently leads to ionic imbalance and membrane depolarization, which, together with excessive glutamate release, triggers excitotoxicity and severe intracellular Ca2+ overload. Concurrent ROS accumulation further promotes neuroinflammation through astrocyte and microglial activation. These interconnected pathological cascades ultimately result in neuronal apoptosis and necrosis, BBB disruption, and cerebral edema. Progressive neuronal loss subsequently contributes to neural circuit remodeling after stroke. Created in https://BioRender.com.

Although initially mobilized as an adaptive survival mechanism, this sustained endocrine overdrive rapidly transitions into a systemic pathological driver. Ultimately, the resulting hypercortisolemic state precipitates a spectrum of secondary complications, directly mediating peripheral immune-metabolic dysregulation, blood–brain barrier breakdown, and the eventual establishment of GR resistance (see Figure 2).

### 4.1 Systemic consequences: metabolic maladaptation and immune dysregulation

Maladaptive hypercortisolemia is recognized as a hallmark of the post-stroke stress response. Elevated serum cortisol levels are positively associated with mortality in patients with AIS. Furthermore, a significant correlation exists between serum cortisol levels and specific markers of the inflammatory response. These markers include body temperature, fibrinogen levels, leukocyte counts, and β-thromboglobulin levels (Slowik et al., 2002).

Metabolically, the physiological response involves an initial central stimulation of ACTH release. This is subsequently followed by feedback inhibition mechanisms that are accompanied by increased adrenal sensitivity. Given that GCs are known to exacerbate neuronal hypoxic injury, their robust release during the hyperacute phase of stroke may intensify the extent of cerebral damage.

Acute ischemic stroke frequently presents with hyperglycemia, clinically referred to as stress-induced hyperglycemia (SIH). GCs are considered a major contributor to this response (Clore and Thurby-Hay, 2009). During the acute phase of stroke, activation of the HPA axis results in elevated circulating levels of GCs and catecholamines. Mechanistically, this hormonal surge promotes hepatic gluconeogenesis and glycogenolysis while concurrently inhibiting peripheral glucose uptake, thereby contributing to the development of SIH (Yao et al., 2023; Gulyaeva et al., 2021). The hyperglycemic state exacerbates cerebral ischemic injury and expands the extent of tissue necrosis. It is associated with a post-injury hypermetabolic state, increased lactate accumulation, and intracellular acidosis. These metabolic disturbances further impair cellular energy metabolism within the ischemic territory, potentially enlarging infarct volume and worsening clinical outcomes (Yao et al., 2023). These detrimental effects are particularly pronounced in individuals with comorbid conditions such as diabetes mellitus. In diabetic patients, pre-existing dysregulation of the HPA axis and elevated basal cortisol levels are frequently observed. Kim et al. demonstrated that HPA axis activation exacerbates ischemic stroke pathology in diabetic models (Kim et al., 2022). Administration of metyrapone—an inhibitor of GCs synthesis—attenuated IL-6 expression and reduced infarct size in the ischemic brains of diabetic mice.

The HPA axis initially functions as a key regulator of the host immune response by modulating inflammatory mediators. This balance enhances infection resistance while preventing excessive immune activation (Silverman and Sternberg, 2012). However, following the onset of IS, the ensuing cytokine surge can disrupt GC-mediated negative feedback regulation of the HPA axis. This disruption sustains pathologically elevated GC levels, establishing a self-perpetuating cycle between inflammation and neuroendocrine dysfunction (Bellavance and Rivest, 2014).

In summary, the HPA axis stress response following IS initially functions to maintain systemic energy homeostasis during the acute phase. However, sustained and excessive activation post-injury contributes to stress-induced hyperglycemia, metabolic maladaptation, and immune dysregulation, ultimately leading to a systemic neuroendocrine–metabolic–immune imbalance. Consequently, strategies aimed at restoring HPA axis stability may represent a promising therapeutic approach for improving overall outcomes in IS.

### 4.2 Secondary brain injury: BBB disruption and hemorrhagic transformation

The BBB, primarily formed by cerebral microvascular endothelial cells, constitutes a highly selective interface between the systemic circulation and the CNS (Neuwelt et al., 2008). While physiological GC signaling has been shown to enhance BBB integrity in murine brain microvascular endothelial cells (Förster et al., 2005), sustained HPA axis hyperactivation following IS is associated with disruption of the NVU and subsequent BBB breakdown (Salvador et al., 2014).

Several interrelated mechanisms contribute to this process. Elevated GC levels, in concert with oxidative stress, are associated with reduced expression of key tight junction proteins, particularly claudin-5 and occludin. This reduction in tight junction integrity leads to increased paracellular permeability (Liu et al., 2012; Yuan et al., 2020). In parallel, HPA axis–associated stress hyperglycemia promotes the accumulation of advanced glycation end products (AGEs) (Kuzan et al., 2024; Sapkota et al., 2021). Engagement of AGEs with their receptor (RAGE) on endothelial cells promotes the upregulation of matrix metalloproteinase-9 (MMP-9) (Ning et al., 2012). Activated MMP-9 subsequently degrades basal lamina components, including type IV collagen and laminin, thereby contributing to structural compromise of the BBB (Rosenberg, 2002). Concurrently, elevated GC levels can directly affect perivascular astrocytes, inducing retraction of astrocytic end-feet and mislocalization of aquaporin-4 (AQP4). These structural alterations disrupt astrocyte–endothelial coupling, further destabilize the NVU, and exacerbate vasogenic edema.

Although recanalization therapies, such as intravenous thrombolysis and mechanical thrombectomy, have revolutionized the management of AIS, they remain associated with a clinically significant risk of hemorrhagic transformation (HT) (Liu et al., 2022). The pathogenesis of HT is largely attributed to the post-ischemic cascade, in which an exaggerated inflammatory response disrupts BBB integrity, leading to vasogenic edema and subsequent erythrocyte extravasation (Qiu et al., 2021). Excessive GC exposure may increase the susceptibility of cerebral microvessels to ischemia–reperfusion injury (IRI). Such exposure may impair the restoration of selective BBB permeability following recanalization, potentially rendering cerebral vessels more vulnerable to hemodynamic stress associated with rapid reperfusion. Consequently, endothelial injury, inflammatory cell infiltration, and matrix degradation—initially triggered by ischemia—may be further exacerbated, thereby contributing to the structural and molecular basis of HT (Fassbender et al., 1994; Sorrells and Sapolsky, 2007; Montagne et al., 2015).

In summary, in AIS patients undergoing recanalization therapy, the HPA axis-mediated hypercortisolemic state may indirectly increase the risk of HT by attenuating BBB repair capacity and potentiating reperfusion-associated inflammatory and proteolytic cascades.

### 4.3 Chronic dysregulation: structural remodeling and functional deficits

In the chronic phase of IS, the HPA axis may fail to return to baseline physiological levels, resulting in sustained dysregulation. This persistent hyperactivity is thought to be associated with ischemic injury to key limbic structures that normally exert inhibitory control over the PVN.

Ion channels and receptors within the hypothalamic PVN play a pivotal role in regulating neuronal activity and neuroendocrine function. Emerging evidence suggests that molecular alterations within the PVN may contribute to systemic complications. Sun et al. observed enhanced hypothalamic neuronal activity, particularly within the PVN, in MCAO rats. Microinjection of MK-801 to inhibit NMDARs within the PVN reduced blood pressure and renal sympathetic nerve discharge (RSND) (Sun et al., 2024). Furthermore, reduced hydrogen sulfide (H2S) levels were implicated in altered sympathetic outflow following cerebral infarction. Collectively, these findings suggest a mechanistic association between PVN dysregulation and post-stroke hypertension. Additionally, Wang et al. identified ion channels in the PVN, such as ASIC1a, as potential therapeutic targets for modulating neuronal excitability under ischemic stress (Wang et al., 2022). Notably, the PVN has also been implicated in the regulation of specific motor functions. Yuan et al. demonstrated that electroacupuncture (EA) at the Lianquan acupoint (CV23) may improve swallowing function by modulating excitatory neurons in the PVN (Yuan et al., 2022). Using viral tracing techniques, they further demonstrated that the beneficial effects of EA on post-stroke dysphagia were attenuated following inhibition of PVN neurons.

Stroke lesions may disrupt HPA-inhibitory regions such as the or medial temporal lobe, resulting in sustained HPA axis activation. Ischemic injury to the hippocampus may result in diminished inhibitory control over the PVN, thereby exacerbating hypercortisolemia. Chronic GC exposure has been associated with reduced dendritic spine density in the PFC, suppression of hippocampal neurogenesis, and amygdalar dysfunction. Lesions in these regions may disrupt negative feedback projections to the PVN and are closely associated with the development of post-stroke memory and cognitive deficits (Sapolsky, 2000).

Chronic exposure to supraphysiological GC levels has been associated with maladaptive remodeling of the neuronal cytoskeleton, which may represent an important pathological substrate for post-stroke functional impairment. Beyond structural alterations, chronic HPA axis hyperactivity also reshapes neuroendocrine signaling networks. Under these pathophysiological circumstances, persistently elevated GC levels fail to effectively suppress the secretion of CRH and ACTH through traditional negative feedback loops, culminating in a maladaptive, chronic hyperactivity of the HPA axis and impaired neuroendocrine homeostasis,which is recognized as GR resistance (Herman et al., 2016). In alignment with the structural and functional breakdown illustrated in Figure 2 (and elaborated in Section 3), this resistance is characterized by disrupted receptor function stemming from chronic neuroinflammatory signaling and prolonged ligand exposure (Zhanina et al., 2022). The post-stroke aberrant release of pro-inflammatory cytokines acts as a primary driver of this state, directly interfering with GR transcriptional activity and diminishing downstream receptor sensitivity. Furthermore, this prolonged activation of glucocorticoid pathways is inherently associated with a compensatory downregulation of NR3C1 expression, thereby yielding diminished receptor availability and compromised negative feedback responsiveness within the remodeled cerebral circuits (McEwen et al., 2016). Crucially, this neuroendocrine breakdown directly perpetuates the localized neuronal excitation/inhibition (E/I) imbalance initially triggered in the acute phase (Chen et al., 1998). Under physiological baselines, balanced glutamatergic and GABAergic signals strictly gate HPA axis reactivity. However, the establishment of GR resistance dismantles this gating mechanism. The loss of functional GR signaling severely compromises astrocytic glutamate clearance and exacerbates GABAergic interneuron deficits, thereby driving sustained glutamate-mediated excitotoxicity within these remodeled circuits (Herman et al., 2004). Specifically, the AVP precursor (pre-proAVP) is synthesized in hypothalamic neurosecretory neurons and transported axonally to the neurohypophysis for processing into mature AVP. AVP has been implicated in the progression of stroke-induced injury (Chojnowski et al., 2023), where it may act synergistically with CRH to sustain the hypersecretion of ACTH and GCs. Concurrently, CRH overexpression has been reported to reduce CREB phosphorylation in the hippocampal CA1 region, possibly through modulation of BDNF signaling pathways, ultimately contributing to impaired synaptic plasticity (Tang et al., 2026).

**Figure 3:**
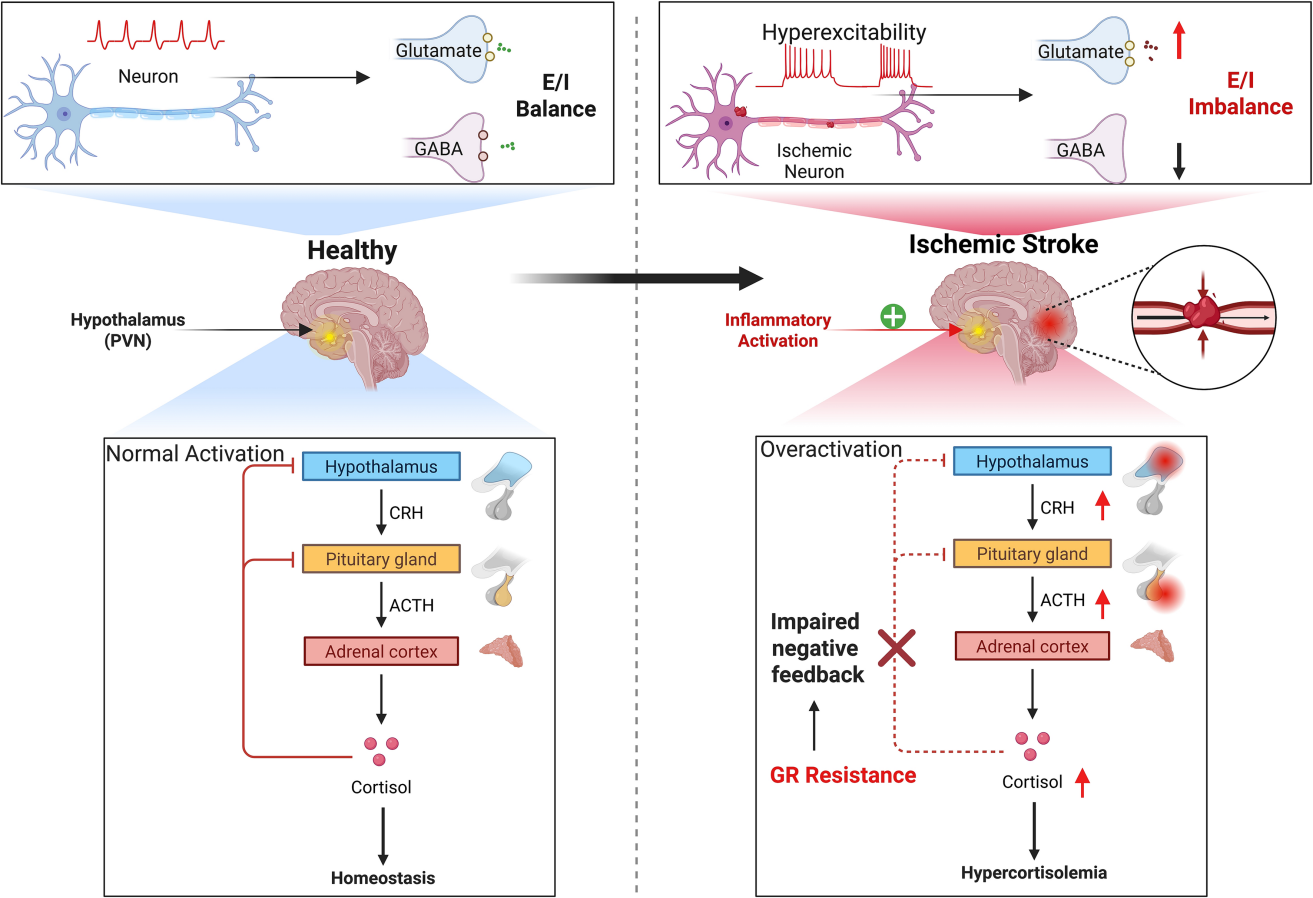
Schematic diagram of neural excitability, HPA axis regulation, and glucocorticoid dynamics in health and during ischemic stroke. Under normal conditions (left), neuronal E/I signals are balanced, and the HPA axis maintains homeostasis via intact negative feedback of cortisol. After ischemic stroke (right), neuronal ischemia and inflammatory activation disrupt this equilibrium. Neurons exhibit hyperexcitability with elevated glutamate and reduced GABA, causing E/I imbalance. Simultaneously, the HPA axis is overactivated. GR resistance impairs the negative feedback loop, leading to the pathological accumulation of CRH, ACTH, and cortisol. Created in https://BioRender.com.

Functional recovery following stroke requires neuronal salvage, tissue regeneration, and neural network reorganization. The re-establishment of HPA axis homeostasis may represent an integral component of this process. Given the restorative potential of neuroplasticity, strategies aimed at normalizing HPA axis function may serve as a promising adjunct to neurorehabilitation. The transition from physiological homeostasis to stroke-induced dysregulation is summarized in Figure 3.

## 5 The pathogenic role of HPA axis dysregulation in post-stroke complications

### 5.1 HPA axis and post-stroke depression

PSD represents a prevalent neuropsychiatric sequela of IS, affecting approximately 30–50% of survivors (Li et al., 2026; Mansour et al., 2025). Its pathophysiology is intrinsically linked to the depletion of central monoaminergic neurotransmitters—specifically 5-HT, DA, and NE—as well as the bidirectional dysregulation of the inflammation–HPA axis network. Wang et al. reviewed 18 studies and identified that post-IS HPA axis dysfunction manifests as elevated basal cortisol and a flattened diurnal rhythm, specifically the loss of the morning cortisol peak (Wang et al., 2024). Notably, admission hypercortisolemia has been proposed as a putative biomarker for post-IS cognitive and emotional outcomes, with elevated levels predicting a higher likelihood of cognitive decline and depression in survivors. Such secretory irregularities are attributed to impaired HPA axis dynamics. Furthermore, Shapero et al. demonstrated a positive correlation between the severity of depressive symptoms and the magnitude of HPA axis activation (Shapero et al., 2019).

The association between HPA axis dysfunction and PSD is thought to involve complex neuro–immune–endocrine interactions. Post-injury HPA axis dysregulation has been associated with alterations in inflammatory cytokine profiles (Hassamal, 2023). Following AIS, peripheral and central pro-inflammatory cytokines (e.g., IL-1, TNF-*α*, CRP) increase markedly during the acute phase. These mediators may access the hypothalamus through a compromised BBB or via circumventricular organs, potentially contributing to sustained HPA axis activation (Pawluk et al., 2025). This inflammatory milieu has been shown to upregulate the rate-limiting enzyme indoleamine 2,3-dioxygenase (IDO). IDO activation diverts tryptophan metabolism toward the kynurenine pathway, thereby reducing tryptophan availability for serotonin synthesis. This cascade may consequently reduce 5-HT synthesis and release, ultimately lowering central 5-HT availability (Kronenberg et al., 2014). In the context of PSD after AIS, HPA axis reactivity may also be influenced by the levels and functional status of serotonin, dopamine, and BDNF (Cameron, 2006). These factors are key components of mood-regulating neural circuits and have been implicated in the pathophysiology of major depressive disorder (MDD). Furthermore, emerging evidence suggests that microRNAs (miRNAs) may influence depressive states, in part by modulating HPA axis function (Li et al., 2025).

Sustained HPA axis hyperactivation is characterized by persistent hypercortisolemia. This condition has been associated with reduced expression of hippocampal brain-derived neurotrophic factor (BDNF) and its receptor, TrkB, which may impair regenerative processes and synaptic plasticity. Chronic hypercortisolemia disrupts the functional integrity of the PFC–hippocampus–amygdala emotion-regulation network. This impairment is driven in part by the attenuation of PFC-mediated inhibitory control over amygdalar activity.(Douglass et al., 2023). Zhanina et al. demonstrated that focal cerebral injury is accompanied by an exaggerated inflammatory response, which can interfere with HPA axis regulation (Zhanina et al., 2022). This inflammatory milieu has been associated with elevated hormonal secretion and alterations in the expression and functional properties of corticosteroid receptors within key limbic regions. Consequently, the sensitivity of limbic GRs to negative feedback regulation is directly impaired by these inflammation-related modifications.

Anatomically, stroke lesions may directly compromise HPA-inhibitory regions, such as the PFC or medial temporal lobe. Due to the resulting loss of top-down inhibitory control over the PVN, sustained HPA axis activation is characteristically maintained. This ischemia-induced disinhibition further perpetuates hypercortisolemia. The presence of persistently elevated cortisol levels and vulnerability to delayed secondary hippocampal injury dictates strict clinical oversight. This is driven by the direct linkage of hippocampal dysfunction to post-stroke cognitive impairment, emotional disturbances, and epileptogenesis (Gulyaeva et al., 2021). These changes alter functional connectivity across cortical networks, thereby promoting the development of depression-like phenotypes. And such alterations effectively recalibrate the regulatory set-points of the negative feedback system. In addition, Xu et al. investigated the efficacy of the selective serotonin reuptake inhibitor (SSRI) paroxetine combined with the traditional Chinese medicine (TCM) formula “Xiaoyao San” in 92 PSD patients over 4 weeks (Xu et al., 2026). The study reported significant amelioration of depressive symptoms. This therapeutic efficacy was accompanied by partial restoration of HPA axis homeostasis, suggesting that re-establishing neuroendocrine balance may represent a promising strategy for the management of PSD.

### 5.2 HPA axis and post-stroke anxiety (PSA)

Post-stroke anxiety (PSA) represents a frequent neuropsychiatric sequela, affecting approximately 33% of IS survivors. Notably, the comorbidity rate of PSA and PSD is estimated at 25% (Ignacio et al., 2024). Recent longitudinal studies have identified PSA as an independent predictor of compromised functional independence and increased mortality. These adverse outcomes are thought to be mediated, at least in part, by chronic neuroendocrine stress (Chun et al., 2018; Barugh et al., 2014).

PSA is characterized by physiological hyperarousal, manifesting as sustained tension, heightened vigilance, an exaggerated startle response, and signs of sympathetic hyperactivity such as tachycardia and increased muscle tone (Gu et al., 2024). This clinical presentation is distinct from the hypoactive and withdrawal-related symptoms typically observed in PSD. Nevertheless, the pathogenesis of PSA involves the synergistic interaction between HPA axis activation and sympathetic nervous system (SNS) hyperexcitability (Chen et al., 2026). Amygdala-dependent fear-processing circuitry is characteristically potentiated by elevated GC levels via enhanced basolateral amygdala (BLA) excitability. This pathological shift directly predisposes patients to pathological worry, irritability, and psychomotor agitation (Grace et al., 2022).

Concurrently, marked sympathetic activation operates via the PVN–ventrolateral medulla (VLM)–intermediolateral cell column (IML) pathway to increase norepinephrine release. This neurotransmitter release directly drives systemic physiological alterations, including tachycardia, reduced heart rate variability, and blood pressure fluctuations. These somatic manifestations may reinforce interoceptive threat perception, thereby amplifying the patient’s subjective experience of fear, particularly concerning potential clinical deterioration or mortality (Herman et al., 2005; Makovac et al., 2016).

Furthermore, clinical data indicate that patients with anxiety exhibit significantly elevated plasma catecholamine (norepinephrine, epinephrine) concentrations and salivary cortisol levels compared to non-anxious individuals. These biological markers correlate positively with scores on anxiety scales, such as the Hamilton Anxiety Rating Scale (HAMA) (Chen et al., 2021; de Koning et al., 2013). Collectively, these findings support a mechanistic link between PSA and dysregulated neuroendocrine stress responses driven by HPA axis dysfunction.

### 5.3 HPA axis and post-stroke cognitive impairment (PSCI)

Post-stroke cognitive impairment (PSCI) is clinically characterized by memory deficits, executive dysfunction, and attentional impairments. The pathogenesis of PSCI is thought to be intrinsically associated with chronic, cumulative CNS injury resulting from neuro-immune-endocrine imbalances (El Husseini et al., 2023). Epidemiological data indicate that approximately 34.2% of IS survivors develop PSCI within the first 6 months post-injury (Huang et al., 2022; Dong et al., 2021).

Altered circulating levels of inflammatory biomarkers, such as C-reactive protein (CRP), IL-6, and IL-10, have been correlated with the development of PSCI (Zhang and Bi, 2020). Moreover, sustained dysregulation of the inflammation–HPA axis network may contribute to the pathological progression of VCI. Structurally, this process manifests as the exacerbation of white matter lesions and progressive global or regional cerebral atrophy. Consequently, these neuroanatomical alterations compromise activities of daily living (ADL), leading to impairments in essential functional capacities.

Circulating inflammatory cytokines can trigger the activation of central microglia, promoting the release of neurotoxic mediators such as reactive oxygen species (ROS), nitric oxide (NO), and proteases. This neurotoxic cascade induces structural damage to neurons within the hippocampal CA1 region and the PFC, manifesting as synaptic loss and dendritic atrophy (Sulhan et al., 2020; Huang et al., 2022). In parallel, chronic exposure to a hypercortisolemic milieu downregulates the expression of key proteins governing synaptic plasticity, including PSD-95, Synapsin-1, and BDNF, thereby impeding synaptogenesis and neural repair processes (McEwen et al., 2016). Furthermore, dysregulation of the hippocampal MR/GR ratio restricts neural progenitor cell proliferation within the dentate gyrus. These lesions ultimately manifest as clinical deficits in declarative memory and executive function.

The causal involvement of stress hormones in PSCI is further corroborated by pharmacological blockade studies, which highlight the critical crosstalk between the HPA axis and the sympathetic nervous system (SNS). Milot et al. demonstrated that ischemia constitutes a potent physiological stressor. Following stroke, the sensitivity and responsiveness of stress hormone systems are upregulated over prolonged intervals, contributing to hippocampal neuronal injury and subsequent memory deficits. Notably, these memory impairments were attenuated not only by the GC synthesis inhibitor metyrapone but also by the *α*-adrenergic agonist clonidine. Conversely, administration of yohimbine exacerbated these deficits. This evidence substantiates the hypothesis that the synergistic hyperactivity of GCs and norepinephrine represents a modifiable driver of post-stroke memory dysfunction (Milot and Plamondon, 2011).

It is noteworthy that these neuropsychiatric complications often intertwine, exacerbating one another (Ruthmann et al., 2025). Patients presenting with comorbid depression, anxiety, and cognitive impairment exhibit significantly diminished quality of life scores and higher mortality rates within the first year post-stroke compared to those without such complications (Stein et al., 2018). Mechanistically, depressive symptoms lead to reduced social engagement and cognitive activity, thereby accelerating cognitive decline. Conversely, functional decline associated with cognitive impairment may induce feelings of inadequacy and helplessness, further deepening depressive states. Additionally, anxiety amplifies fear and uncertainty regarding prognosis, leading to reduced treatment adherence—such as resistance to rehabilitation protocols—thereby impeding neurological recovery.

### 5.4 HPA axis and stroke-associated pneumonia (SAP)

The incidence of stroke-associated pneumonia (SAP) in stroke units has been reported to range up to approximately 30% (Hannawi et al., 2013; Westendorp et al., 2011). Although recent data suggest a declining trend in post-stroke infection rates, SAP remains the most prevalent infectious complication and an important contributor to mortality following IS (Awere-Duodu et al., 2024). SAP is not merely a mechanical consequence of aspiration secondary to dysphagia; rather, it is increasingly recognized as a clinical manifestation of stroke-induced immunosuppression syndrome (SIDS). Following IS, the immune system shifts toward a state of systemic suppression, rendering patients more susceptible to opportunistic pathogens (Meisel et al., 2005). SIDS is characterized by acute lymphopenia and splenic atrophy. Phenotypically, this is reflected by a marked reduction in both the number and functional competence of peripheral T cells, B cells, and natural killer (NK) cells (Offner et al., 2009). The pathogenesis of this immunosuppressive state involves the coordinated activation of the HPA axis and the SNS, with the HPA axis considered a central mediator of post-ischemic immunodepression (Prass et al., 2003).

IS triggers increased GCs secretion, which directly induces lymphocyte apoptosis through GRs binding and suppresses human leukocyte antigen-DR (HLA-DR) expression on monocytes (Dirnagl et al., 2007). Concurrently, catecholamines released by sympathetic nerves cooperate with the HPA axis to drive a phenotypic shift of helper T cells from the pro-inflammatory Th1 toward the anti-inflammatory Th2 subtype. This specific suppression of cellular immunity markedly enhances susceptibility to commensal organisms, particularly translocated gut bacteria (Lowrance et al., 2016; Stanley et al., 2016).

Additionally, clinical data demonstrate that patients who develop SAP exhibit significantly elevated serum levels of cortisol and norepinephrine compared with those without infection. Moreover, low monocyte HLA-DR expression serves as an independent biomarker for predicting post-stroke infections (Harms et al., 2008; Katan et al., 2009). These findings validate HPA axis-mediated neuroendocrine dysregulation as a critical driver underlying the collapse of immune barriers and secondary infections following stroke.

### 5.5 HPA axis changes after IS affect circadian rhythms

As previously discussed, GCs regulate circadian rhythms under physiological conditions. This rhythmicity depends on transcription-translation feedback loops (TTFLs) of core clock genes in both SCN neurons and adrenocortical cells (Mohawk et al., 2012). Experimental studies demonstrate that ischemic stroke downregulates BMAL1 and REV-ERBα expression in peripheral tissues, thereby disrupting steroidogenic pathways (Pu et al., 2025; Henein et al., 2022). Consequently, the adrenal cortex becomes hypersensitive to stress signals, resulting in aberrant temporal patterns of cortisol secretion that persist even during phases that should represent physiological nadirs.

Concurrently, the massive release of pro-inflammatory cytokines following AIS directly stimulates the PVN, bypassing SCN regulation and thereby causing circadian misalignment of cortisol peaks. Clinically, this manifests as stress-induced hypertension (Dumbell et al., 2016). Quantitative studies indicate that nocturnal cortisol elevation exceeding 50% of morning levels during this phase serves as an independent predictor of early neurological deterioration and mortality (Barugh et al., 2014).

During the chronic phase of IS, the amplitude of the diurnal cortisol curve becomes significantly attenuated. This phenomenon likely relates to GCs resistance in the hippocampus, which impairs negative feedback loops. Such chronic disruption of circadian rhythmicity suppresses hippocampal neurogenesis and synaptic plasticity, establishing a pathological link with PSD and VCI (Villa et al., 2018).

Progressive exercise training reduces thalamic CRH mRNA expression and restores hippocampal neurogenesis. Acupuncture also ameliorates HPA axis function through modulation of the neuro-endocrine-immune network. Electroacupuncture (EA) at meridian points effectively downregulates serum corticosterone, hypothalamic CRF mRNA, and pituitary ACTH mRNA expression, while upregulating hypothalamic GR mRNA—effects that likely contribute to its protective role against CIRI (Cai et al., 2009).

## 6 Conclusion

This review positions the HPA axis as a pivotal nexus in ischemic stroke pathophysiology, extending beyond its classical role in acute stress responses. It emerges as a central orchestrator linking cerebral ischemia to systemic metabolic dysregulation, immune paralysis, and BBB disruption. Through the integration of circadian control, GRs signaling, and multi-organ crosstalk, HPA axis dysfunction underpins both neurological damage and extra-cerebral complications after stroke.

These temporal dynamics reveal dichotomous HPA axis effects across disease phases. Acutely, glucocorticoid surge constitutes an evolutionarily conserved mechanism for energy mobilization; yet aberrant amplification precipitates secondary injury cascades, encompassing stress hyperglycemia, immunosuppression, and hemorrhagic transformation. With chronicity, sustained exposure erodes hippocampal and prefrontal inhibitory circuits, crippling central negative feedback. This failure marks the pathological pivot from adaptive response to neurotoxic driver. Prolonged dysregulation suppresses neurotrophic signaling and arrests endogenous neurogenesis, erecting biological barriers to functional recovery. The resulting maladaptive plasticity fuels synergistic deterioration of PSD, anxiety, and cognitive impairment.

The disruption of circadian cortisol rhythmicity may serve as an early prognostic biomarker after ischemic stroke. We therefore propose that therapeutic strategies prioritize HPA feedback restoration and neuroendocrine-immune recalibration. Targeting this axis offers dual potential: acute neuroprotection for ischemic tissue and sustained facilitation of neurorehabilitation. This approach holds promise for holistic recovery and the prevention of long-term post-stroke morbidity.
